# Oral prednisolone for acute lower respiratory tract infection in clinically unrecognised asthma: an exploratory analysis of the Oral Steroids for Acute Cough (OSAC) randomised controlled trial

**DOI:** 10.3399/bjgpopen20X101099

**Published:** 2020-11-04

**Authors:** Sean Hawkey, Grace J Young, Paul Little, Michael Moore, Alastair D Hay

**Affiliations:** 1 Center for Academic Primary Care, National Institute for Health Research (NIHR) School for Primary Care Research, Bristol Medical School, University of Bristol, Bristol, UK; 2 Department of Population Health Sciences, Bristol Medical School, University of Bristol, Bristol, UK; 3 Bristol Trials Centre, Bristol Medical School, University of Bristol, Bristol, UK; 4 Primary Care and Population Science, NIHR School for Primary Care Research, Aldermoor Health Centre, University of Southampton, Southampton, UK

**Keywords:** asthma, respiratory tract infections, randomised controlled trial, primary healthcare, general practice

## Abstract

**Background:**

Acute lower respiratory tract infection (ALRTI) is often treated in primary care with antibiotics. The recent Oral Steroids for Acute Cough (OSAC) randomised controlled trial (RCT) showed corticosteroids were not an effective alternative in adults without a diagnosis of asthma with ALRTI.

**Aim:**

To investigate if corticosteroids are beneficial for ALRTI in patients with unrecognised asthma.

**Design & setting:**

An exploratory analysis was undertaken of the primary care OSAC trial.

**Method:**

A subgroup analysis was performed in patients who responded ‘yes’ to the following International Primary Care Airways Group (IPCAG) question: did you have wheeze and/or at least two of nocturnal cough or chest tightness or dyspnoea in the past year. Sensitivity analyses were carried out on those who answered ‘yes’ to wheeze and at least two of the nocturnal symptoms. The primary outcomes were as follows: duration of cough (0–28 days, minimum clinically important difference [MCID] of 3.79 days) and mean symptom severity score (range 0–6; MCID 1.66 units).

**Results:**

In total, 40 (10%) patients were included in the main analysis: mean age 49 years (standard deviation [SD] = 17.9), 52% male. Median cough duration was 3 days in both prednisolone (interquartile range [IQR] = 2–6 days) and placebo (IQR = 1–6 days) groups (adjusted hazard ratio [HR] = 1.10; 95% confidence interval [CI] = 0.47 to 2.54; *P* = 0.83), equating to 0.24 days longer in the prednisolone group (95% CI = 1.23 days shorter to 2.88 days longer). Mean symptom severity difference was –0.14 (95% CI = –0.78 to 0.49; *P*=0.65) comparing prednisolone with placebo. Similar findings were found in the sensitivity analysis.

**Conclusion:**

No evidence was found to support the use of corticosteroids for ALRTI in patients with clinically unrecognised asthma. Clinicians should not use the IPCAG questions to target oral corticosteroid treatment in patients with ALRTI.

## How this fits in

Corticosteroids are an increasingly used alternative to antibiotics for ALRTI in some countries. The recently published OSAC randomised trial was the first to investigate the effectiveness of oral steroids in adults without a diagnosis of asthma with ALRTI, and found no evidence to support their use. This exploratory analysis was conducted to see if participants with unrecognised asthma (at the time of randomisation) experienced a better response to oral corticosteroids than the remainder of the study sample (those who did not have unrecognised asthma). This study shows they did not. If they had, it would have been important for two reasons. First, primary care clinicians might have wished to use the British Thoracic Society (BTS) approved questionnaire (used to identify those with unrecognised asthma) to identify those who would benefit the most, and second, it would have provided an important ‘signal’ that the research community might have wished to confirm in future research. In the meantime, clinicians should not use the IPCAG questions to target oral corticosteroid treatment in patients with ALRTI.

## Introduction

ALRTI is one of the most common conditions seen in primary care,^1–3^ accounting for 60% of antibiotic prescribing in general practice^2^ and costing the NHS an estimated 190 million GBP each year.^4^ This not only places a significant financial burden on the NHS, but also contributes to the worldwide problem of emerging antimicrobial resistance.

There is evidence to suggest the effectiveness of antibiotics in ALRTI is limited. A European trial of amoxicillin found that when pneumonia was not suspected, antibiotics did not alter symptom duration or severity.^5^ This was also the case for those deemed at high risk; that is, smokers, and those with green sputum, fever, abnormal lung signs, or known lung disease.^6^ Similar findings were reported in a Cochrane review of nine trials including >750 patients with acute bronchitis.^7^

There is considerable overlap between ALRTI and acute asthma. Both conditions can present with obstructive symptoms associated with reduced forced expiratory volume (FEV).^8,9^ At a cellular level, ALRTI and asthma both cause airway inflammation and transient airway hyper-responsiveness.^8,9^ Although corticosteroids are the mainstay of treatment in asthma,^10^ and are reported to be effective for community-acquired pneumonia treated in hospital,^11^ there has been an absence of evidence for ALRTI in primary care. A systematic review investigating inhaled corticosteroids found insufficient evidence to recommend their use,^12^ and the recently conducted OSAC randomised trial found that oral corticosteroids did not reduce the duration of moderately bad or worse cough, or symptom severity in adults without a diagnosis of asthma with ALRTI.^13^

However, corticosteroids could be more beneficial in those who have unrecognised asthma, or are at risk of developing asthma. Asthma is both underdiagnosed and under-reported, particularly in adult and older populations.^14–18^ A Danish study found 67% of an ‘asthmatic cohort’ (defined by respiratory symptoms and airway hyper-responsiveness to methacholine, peak flow variability >20% or ≥500 ml increase in FEV_1_ after bronchodilator challenge) did not have a formal diagnosis of asthma in their medical records,^14^ with similar results seen in a Dutch^15^ and an Australian study.^16^

Given the theoretical benefit of oral corticosteroids in people with asthma presenting with ALRTI and the underdiagnosis of asthma in the general population, this exploratory analysis of the OSAC trial sought to investigate whether oral corticosteroids reduced the severity and duration of symptoms in a subgroup of patients with clinically unrecognised asthma. If shown to be the case, further research would need to replicate the finding, which could then inform the selection of patients with ALRTI most likely to benefit from oral corticosteroids.

## Method

### Summary of the OSAC trial

The study details are reported fully in the original OSAC article.^13^ To summarise, participants were recruited from 54 GP practices between July 2013 and October 2014. Patients were eligible if aged ≥18 years, presenting with an acute (≤28 days) cough with at least one lower respiratory tract infection (LRTI) symptom — phlegm, chest pain, wheezing, or shortness of breath — in the last 24 hours, and not requiring immediate antibiotics. Patients were excluded if they had: chronic pulmonary disease; received any asthma medication in the past 5 years; met the National Institute for Health and Care Excellence (NICE) criteria for severe infection or complications;^2^ required same-day hospital admission; or required same-day antibiotics.

Participants were asked to take two tablets daily for 5 days (prednisolone 20 mg or placebo), starting on the day of consultation, reflecting the dose and duration used in acute asthma. Randomisation was concealed and recruiting clinicians, participants, and trial team members were masked to treatment allocation until data analyses were complete. Participants were asked to report the presence and severity of symptoms using a validated^19^ diary used in previous trials.^4,5^ Symptoms were measured using a zero to six scale, shown to be sensitive to change,^4^ from zero (no problem) to three (moderately bad) and up to six (as bad as it could be). All symptoms were measured daily, with twice daily peak expiratory flow, for 28 days or until symptom resolution.

### Defining clinically unrecognised asthma

Patients were identified as having clinically unrecognised asthma based on answers to the IPCAG questionnaire (see Supplementary Box S1).^20^ This was developed to estimate the population prevalence of asthma, and includes symptoms with the strongest evidence of their value in diagnosing for asthma (wheeze, nocturnal cough or chest tightness or dyspnoea, dyspnoea on exertion, dyspnoea at rest).^10^ The version used in OSAC (see Supplementary Box S2) specified that participants must *‘* *think about the presence of symptoms in the* *12* *months* *before their current illness started* *'*.

The BTS and Scottish Intercollegiate Guidelines Network (SIGN) guidelines state that isolated symptoms are neither sensitive nor specific for asthma, with symptom combinations more useful.^10^ The presence of wheeze and/or at least two out of three nocturnal symptoms (dyspnoea, chest tightness, and cough) were used for the main analyses as this has been shown to be 80.0% sensitive and 85.9% specific.^21^ In case the main analysis definition was too sensitive, a sensitivity analysis was conducted using a more specific definition, including patients who answered ‘yes’ to the presence of wheeze and at least two out of the three nocturnal symptoms. Based on asthma prevalence from asthma.org.uk,^22^ NHS datasets,^23^ and the Quality and Outcomes Framework (QoF)^24^ it was anticipated 5%–17% of the OSAC cohort would have clinically unrecognised asthma.

### Outcomes and statistical methods

The same pre-defined outcomes from the OSAC trial were used.^13^ The first of two primary outcomes was duration of moderately bad or worse cough, defined as the number of days from randomisation to the last day the cough was scored ≥3, prior to at least two consecutive days scored <3, up to a maximum of 28 days. The second was the mean of the six symptom (cough, phlegm, shortness of breath, sleep disturbance, feeling generally unwell, and activity disturbance) severity scores on days 2–4. As this was an exploratory analysis, the sample size was predetermined by the MCID in both outcomes from the original study;^13^ 3.79 days for duration of cough and 1.66 units for symptom severity.

All analyses were carried out in Stata (version 15.1). Main comparative and sensitivity analyses were conducted as per the OSAC trial to allow comparison with the original study, adjusting for the relevant baseline measure (either prior cough duration or patient-reported illness severity) and centre. Semi-parametric Cox-proportional hazard models were used to analyse the duration of moderately bad or worse cough. HRs were reported comparing instantaneous rate of resolution of cough between prednisolone and placebo groups. Parametric Weibull accelerated failure time (AFT) models were used to present time ratios. Mean symptom severity score from days 2–4 was analysed using linear regression models. Secondary additional adjustment was performed where appropriate, accounting for factors demonstrating imbalance at baseline. These were defined prior to the analysis as a difference of >15% for binary and >0.5 SDs for continuous outcomes; a conservative approach to accommodate for the small sample size. Smoking was also adjusted for, a likely confounding factor, given its link with both asthma and cough.^25^ To test whether the treatment effect differed in the defined subgroups, compared with the rest of the OSAC cohort, interactions were included, which were followed by a likelihood ratio test. See Appendix S1 for details of the secondary outcomes and analyses.

## Results

### Study population and outcome data completeness

In total, 40 (10%) of the 398 patients randomised in the OSAC study met the symptom criteria for the main analysis (wheeze and/or at least two of nocturnal wheeze or cough or dyspnoea), and 21 (5%) met the criteria for the sensitivity analysis (wheeze and at least two of nocturnal wheeze or cough or dyspnoea).

Main analysis group participants had a mean age of 49 (SD = 17.9) years; 52% were male; 25% were current smokers; 78% reported phlegm; 68% shortness of breath; 55% wheezing; and 40% had abnormal peak flow. Baseline characteristics were similar between prednisolone and placebo arms. However, in the prednisolone group, a higher percentage of participants were older, lived with a smoker, reported phlegm, and had an abnormal peak flow than the placebo group (Table 1). Conversely, a lower percentage had a family history of atopy than the placebo group. Compared with the rest of the OSAC cohort, a higher percentage of patients were current smokers and had a family history of atopy.

**Table 1.** Baseline characteristics (main analysis groups)

Characteristics, *n* (%)^a^	**Main analysis group** (*N* = 40)	**Rest of OSAC sample**(*N* = 358)
**Prednisolone**(*n* = 22)	**Placebo**(*n* = 18)
**Centre**			
Bristol	15 (68.2)	11 (61.1)	205 (57.3)
Oxford	3 (13.6)	3 (16.7)	78 (21.8)
Southampton	3 (13.6)	1 (5.6)	41 (11.5)
Nottingham	1 (4.5)	3 (16.7)	34 (9.5)
**Demographics and past medical history**			
Sex, male	13 (59.1)	8 (44.4)	127 (35.5)
Mean age, years (SD)	53.02 (18.1)	43.7 (16.6)	47.2 (15.8)
Median weight, kg (IQR)^b^	78.0 (64.0–95.0)	80.5 (70.0–94.0)	76 (65.0–90.0)
Median height, cm (IQR)^c^	172.5 (162.0–176.0)	171.5 (166.0–175.0)	168 (162.0–175.0)
Ethnic group, White^c^	22 (100)	16 (88.9)	343 (96.1)
**Occupation**			
Employed	14 (63.6)	10 (55.6)	256 (71.5)
Unemployed	3 (13.6)	2 (11.1)	13 (3.6)
Retired	4 (18.2)	5 (27.8)	62 (17.3)
Other	1 (4.5)	1 (5.5)	27 (7.5)
**Median deprivation score (** **IQR** **)** ^d^	18.5 (7.0–28.0)	12.5 (10.0–24.5)	11 (5.0–22.0)
**Smoking status** ^c^			
Current	6 (27.3)	4 (22.2)	59 (16.5)
Past	6 (27.3)	10 (55.6)	102 (28.6)
Never	10 (45.5)	4 (22.2)	196 (54.9)
Lives with smoker^e^	3 (15.0)	6 (35.3)	48 (14.1)
**Received asthma medication** **>** **5** **years** **previously** ^f^	1 (5.0)	2 (11.1)	15 (4.4)
**Personal history of hay fever** ^g^	6 (31.6)	5 (29.4)	76 (22.3)
**Personal history of eczema** ^h^	2 (10.5)	4 (22.2)	50 (14.8)
**Family history asthma** **or** **hay fever** **or** **eczema** ^i^	9 (45.0)	11 (61.1)	129 (38.7)
**Influenza vaccine in last** **12** **months**	6 (27.3)	4 (22.2)	97 (27.1)
**Recruited in winter (** **1** **Oct** **ober** **–** **31** **March)**	14 (63.6)	13 (72.2)	199 (55.6)
**Clinical characteristics and management**			
Median prior duration of cough, days (IQR)	12.0 (7.0–21.0)	14.0 (6.0–21.0)	11.0 (6.0–19.0)
Sputum, symptom <24 hour^c^	15 (68.2)	16 (88.9)	274 (76.8)
Shortness of breath, symptom <24 hour	15 (68.2)	12 (66.7)	252 (70.4)
Wheeze, symptom <24 hour^c^	13 (59.1)	9 (50.0)	164 (45.9)
Chest pain, symptom <24 hour	11 (50.0)	7 (38.9)	167 (46.6)
Median patient-reported illness severity (0–10), (IQR)^j^	6.5 (3.0–7.0)	5.0 (4.0–6.0)	6.0 (4.0–7.0)
Mean pulse rate, bpm (SD)	72.9 (13.0)	76.4 (10.6)	78.1 (12.0)
Mean temperature, °C (SD)	36.6 (0.4)	36.6 (0.5)	36.6 (0.5)
Mean oxygen saturation, % (SD)^c^	97.6 (1.8)	97.8 (0.9)	97.7 (1.2)
Baseline abnormal peak flow^c^	11 (50.0)	5 (27.8)	150 (42.0)
Abnormal respiratory rate	0 (0)	0 (0)	3 (0.8)
Chest recession or prolonged expiration	0 (0)	0 (0)	1 (0.3)
Wheeze or rhonchi, auscultation	2 (9.1)	2 (11.1)	18 (5.0)
Crackles or crepitations, auscultation^h^	2 (9.1)	0 (0)	8 (2.2)
Bronchial breathing	0 (0)	1 (5.6)	1 (0.3)
Taken prescribed β agonist in past 24 hours	2 (9.1)	1 (5.6)	9 (2.5)
Over-the-counter drugs taken for current cough	12 (54.5)	12 (66.7)	243 (67.9)
Given delayed antibiotic script	4 (18.2)	3 (16.7)	40 (11.2)

^a^Unless otherwise stated. ^b^Weight missing for 2 in the rest of the OSAC sample. ^c^Height, ethnicity, smoking status, sputum, wheeze, oxygen saturation, and abnormal peak flow data missing for one in the rest of OSAC sample. ^d^English Index of Multiple Deprivation scores (range 0–100; higher scores indicate higher levels of deprivation). Data missing for two in placebo group and seven in the rest of OSAC sample. ^e^Living with smoker data missing for two in the prednisolone group, one in the placebo group and seventeen in the rest of OSAC sample. ^f^Data on use of asthma medication >5 years previously missing for two patients in the prednisolone group and fifteen in the rest of OSAC sample. ^g^Personal history of hay fever data missing for three in the prednisolone group, one in the placebo group and seventeen in the rest of OSAC sample. ^h^Personal history of eczema data missing for three in the prednisolone group and twenty one in the rest of OSAC sample. ^i^Family history of hay fever, eczema, or asthma data missing for two in the prednisolone group and twenty five in the rest of OSAC sample. ^j^Patient-reported illness severity scores: 0 (completely well) to 10 (extremely unwell). Missing for one in the rest of OSAC sample. ^h^Includes unilateral and bilateral. IQR = interquartile range. OSAC = Oral Steroids for Acute Cough. SD = standard deviation.

For duration of moderately bad or worse cough, data were available for 31 (78%) participants in the main analysis group. Six were not included as they reported an initial cough severity of <3 points (that is, not moderately bad or worse), two withdrew, and one was lost to follow-up. For symptom severity, data were available for 37 (92%) participants.

### Primary outcomes

#### Moderately bad or worse cough

**Figure 1.**
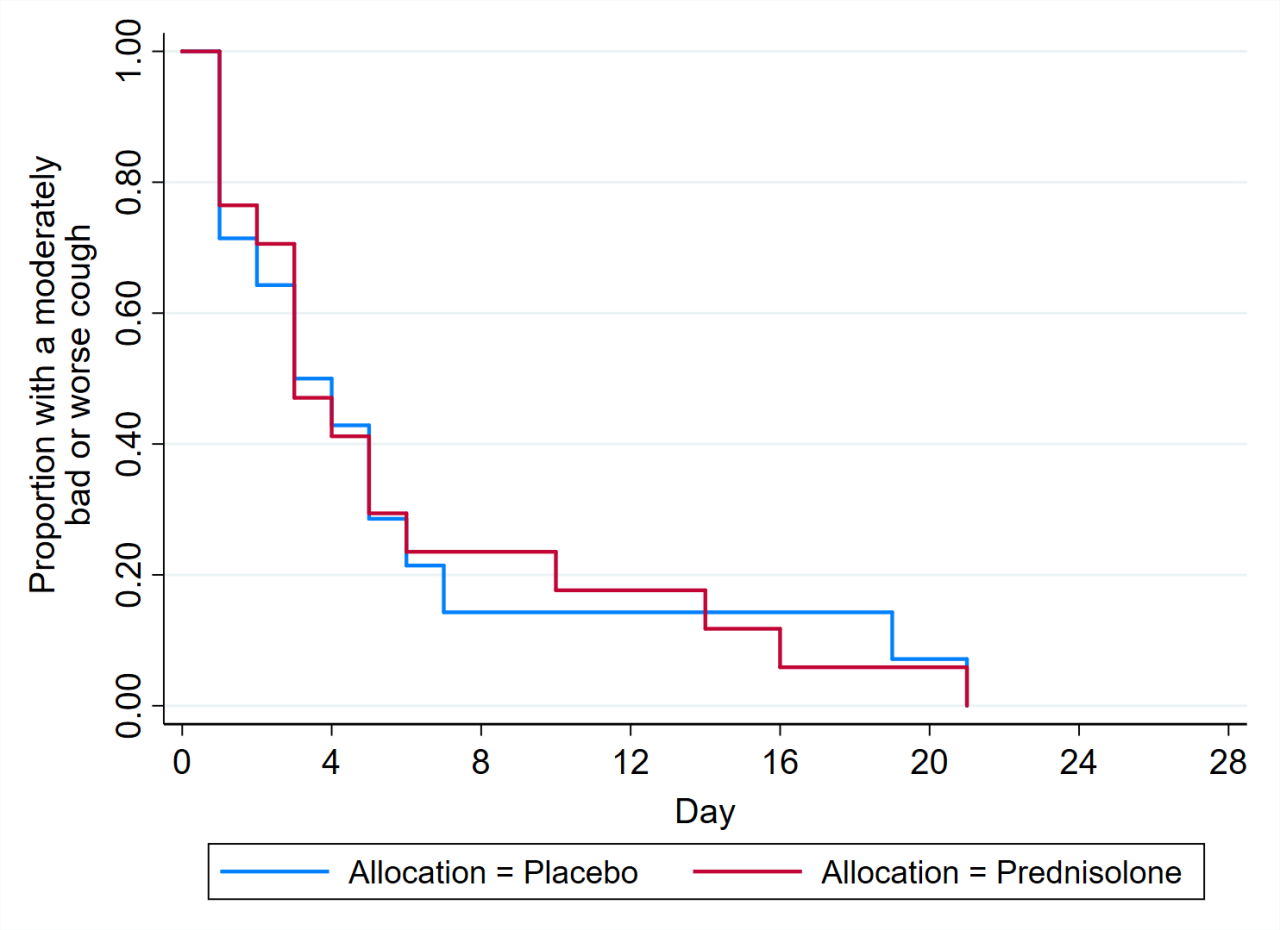
Kaplan–Meier analysis of time to recovery from moderately bad or worse cough (main analysis groups)

In the main analysis group, the median duration of moderately bad or worse cough was 3 days for both prednisolone (IQR = 2–6 days) and placebo arms (IQR = 1–6 days) (Table 2). Kaplan–Meier survival curves showed no clinically important differences between groups (Figure 1). When adjusting for centre and baseline cough duration (pre-specified adjustments in the main trial), the Cox model found a HR of 1.10 (95% CI = 0.47 to 2.54) comparing prednisolone with placebo, with no clear evidence (*P* = 0.83) of a treatment effect (Table 2). The Weibull AFT model time ratio was 1.08 (95% CI = 0.59 to 1.96), showing the time to resolution was 8% (0.24 days) longer (95% CI = 1.23 days shorter to 2.88 days longer) with prednisolone compared with placebo (*P* = 0.79), that is, the 95% CI did not include values that exceeded the pre-specified MCID of 3.79 days. Further adjustment for factors displaying imbalance at baseline (age, lives with smoker, family history of atopy, sputum, and abnormal peak flow) and smoking mildly increased the effect of prednisolone with respect to both the models. Although secondary adjustment did alter the time ratio from 1.08 to 0.77, CIs again remained within the MCID.

**Table 2.** Primary outcomes (main analysis groups)

	**Prednisolone**	**Placebo**	**Prednisolone versus placebo**
*n*	Median (IQR)	*n*	Median (IQR)	HR (95% CI);*P* value	Time ratio^a^ (95% CI); *P* value
**Duration of moderately bad** **or** **worse cough**	17	3 (2–6)	14	3 (1–6)		
Unadjusted					1.01 (0.49 to 2.05); *P* = 0.99	1.03 (0.53 to 2.02); *P* = 0.94
Adjusted for centre and baseline cough duration^b^					1.10 (0.47 to 2.54); *P* = 0.83	1.08 (0.59 to 1.96); *P* = 0.79
Secondary additional adjustment^c^					1.19 (0.39 to 3.75); *P* = 0.75	0.77 (0.38 to 1.57); *P* = 0.47
	***n***	**Mean (** **SD** **)**	***n***	**Mean (** **SD** **)**	**Difference in means (** **95%** **CI** **);** ***P*** **value**	
**Mean symptom severity score** **,** **days** **2** **–** **4** ^d^	21	1.83 (1.05)	16	1.95 (0.87)		
Unadjusted					–0.13 (–0.79 to 0.53); *P* = 0.69	
Adjusted for centre and baseline illness severity					–0.14 (–0.78 to 0.49); *P* = 0.65	
Secondary additional adjustment					0.02 (–1.01 to 1.07); *P* = 0.95	

^a^Time ratio can be interpreted as the relative increase or decrease in time to resolution from moderately bad or worse cough in the prednisolone versus the placebo group. ^b^Baseline measure of duration of cough is prior duration of cough (1–28 days) and of mean symptoms severity score is patient-reported illness severity (range 0–10). ^c^Adjusted for centre, baseline cough duration or illness severity, factors showing imbalance at baseline (age, family history of atopy, smoking status, lives with smoker, sputum, and abnormal peak flow). ^d^See Method section for derivation of mean symptoms severity score (0 [least severe] to 6 [most severe]). HR = hazard ratio. IQR = interquartile range. SD = standard deviation.

#### Day 2–4 symptom severity

Mean symptom severity scores (and residuals) were normally distributed, with a mean symptom severity of 1.83 (SD = 1.05) and 1.95 (SD = 0.87) for the prednisolone and placebo groups, respectively. When adjusting for centre and baseline illness severity, there was a mean symptom severity reduction of -0.14 (95% CI = -0.78 to 0.49; *P* = 0.65) between prednisolone and placebo; that is, the 95% CI did not include values that exceeded the MCID of 1.66. Secondary additional adjustment narrowed the difference in the mean symptom severity between groups.

**Figure 2.**
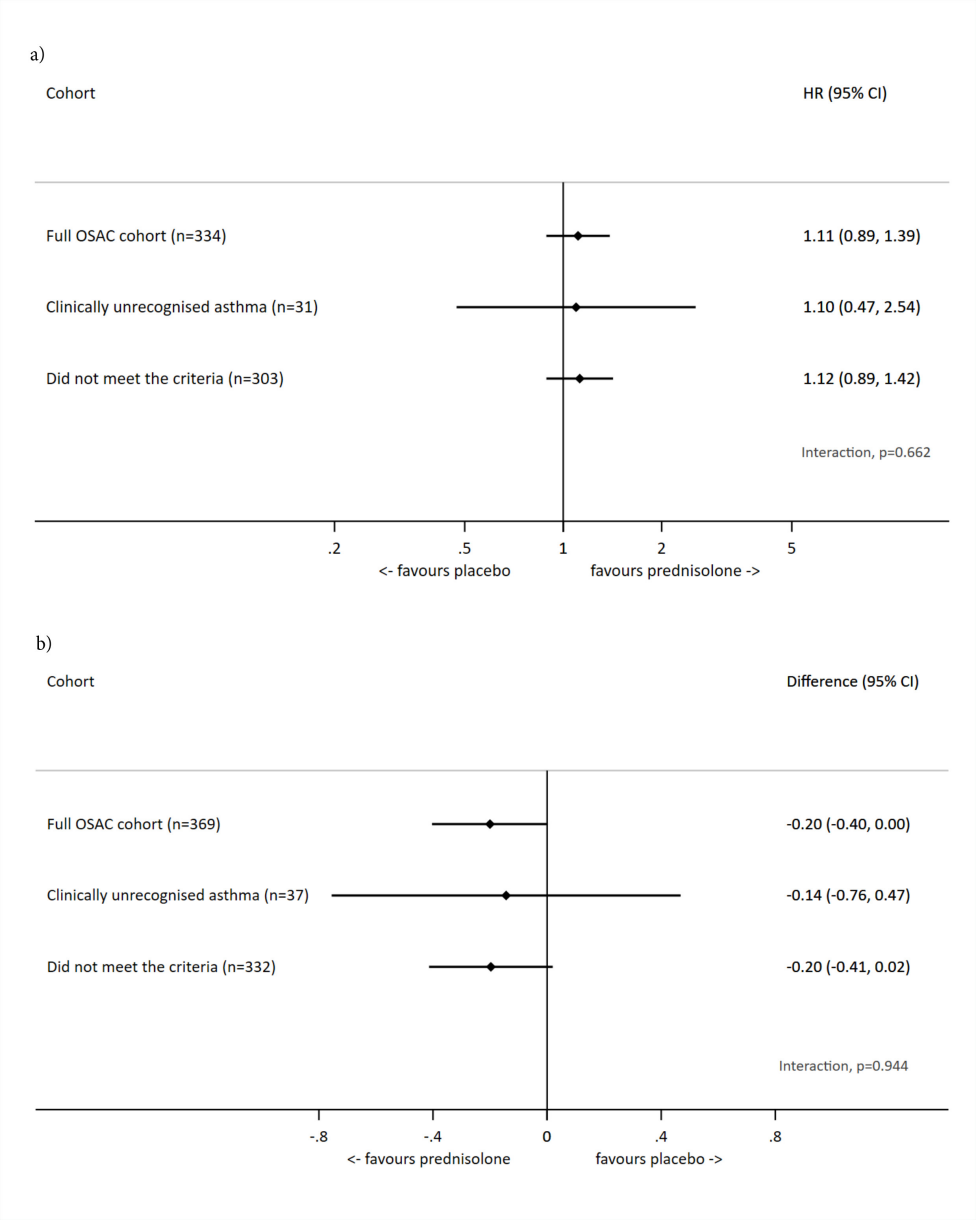
Forest plots of the subgroups for a) duration of moderately bad or worse cough and b) mean symptom severity score. HR = hazard ratio. OSAC = Oral Steroids for Acute Cough

For both primary outcomes, when adding an interaction term for the subgroup, no evidence was found to suggest that there were differential treatment effects seen in those who did and did not meet the criteria for clinically unrecognised asthma (Figure 2a and b). See Appendix 2, Supplementary Table S1, Table S2, and Figure S1 in the supplementary material for the sensitivity analysis results.

### Secondary outcomes

In the main analysis group, after adjusting for baseline symptom, prednisolone had no distinguishable effect on any ALRTI symptoms or duration of abnormal peak flow up to 28 days, or cough up to 56 days (Table 3). There was also no effect on antibiotic use, the proportion of participants stateing they felt better or would use the same medication for similar illnesses in the future or the number of adverse events. Three of the patients in the OSAC trial went on to receive an asthma diagnosis, of which only one met the criteria used in the current analysis. Further details can be found in Appendix S3.

**Table 3.** Secondary outcomes (main analysis groups)

	**Prednisolone** **(*N* = 21)**	**Placebo** **(*N* = 16)**	**Prednisolone versus placebo**
	**Mean A** **U** **C** **(** **SD** **)**		**Difference in mean AUC** **(** **95%** **CI** **);** ***P*** **value**
**Cough**	36.29 (25.89)	34.38 (24.71)	
Unadjusted			1.91 (–15.20 to 19.01); *P* = 0.82
Adjusted for baseline^a^			2.27 (–15.22 to 19.75); *P* = 0.79
**Phlegm**	20.60 (19.97)	30.69 (25.42)	
Unadjusted			–10.09 (–25.23 to 5.04); *P* = 0.19
Adjusted for baseline^b^			–10.35 (–26.18 to 5.48); *P* = 0.19
**Shortness of breath**	12.45 (16.13)	17.56 (19.79)	
Unadjusted			–5.11 (–17.10 to 6.88); *P* = 0.39
Adjusted for baseline^b^			–3.64 (–15.56 to 8.29); *P* = 0.54
**Wheeze**	15.12 (16.4)	16.13 (18.93)	
Unadjusted			–1.01 (–12.82 to 10.80); *P* = 0.86
Adjusted for baseline^b^			–2.10 (–13.75 to 9.54); *P* = 0.72
**Blocked** **or** **runny nose**	15.67 (20.78)	20.15 (25.66)	
Unadjusted			–4.49 (–19.99 to 11.00); *P* = 0.56
Adjusted for baseline^b^			–4.24 (–19.83 to 11.34); *P* = 0.58
**Chest pain**	10.33 (14.50)	5.91 (8.52)	
Unadjusted			4.43 (–3.85 to 12.71); *P* = 0.29
Adjusted for baseline^b^			5.07 (–2.74 to 12.88); *P* = 0.17
**Feve** **r**	3.48 (6.36)	5.53 (14.08)	
Unadjusted			–2.06 (–9.06 to 4.95); *P* = 0.56
Adjusted for baseline^b^			–2.91 (–9.25 to 3.43); *P* = 0.36
**Muscle ache**	13.74 (24.87)	8.31 (14.46)	
Unadjusted			5.43 (–8.75 to 19.60); *P* = 0.44
Adjusted for baseline^b^			5.84 (–8.45 to 20.13); *P* = 0.41
**Headache**	11.64 (24.67)	6.88 (15.84)	
Unadjusted			4.77 (–9.61 to 19.14); *P* = 0.51
Adjusted for baseline^b^			4.91 (–9.54 to 19.36); *P* = 0.50
**Sleep disturbance**	20.29 (24.84)	17.94 (24.07)	
Unadjusted			2.35 (–14.17 to 18.86); *P* = 0.78
Adjusted for baseline^b^			3.23 (–13.67 to 20.12); *P* = 0.70
**Feeling unwell**	19.86 (27.79)	17.19 (22.54)	
Unadjusted			2.67 (–14.62 to 19.96); *P* = 0.76
Adjusted for baseline^b^			1.29 (–15.35 to 17.93); *P* = 0.88
**Activity disturbance**	10.14 (13.16)	10.59 (20.64)	
Unadjusted			–0.45 (–11.75 to 10.85); *P* = 0.94
Adjusted for baseline^b^			–0.15 (–11.56 to 11.27); *P* = 0.98
	**Median duration (** **IQR** **)**		**HR** **(** **95%** **CI** **);** ***P*** **value**
**Duration of mod** **erate** **-bad** **or** **worse cough (censored at** **56** **days** **)** ^c^	3 (2–6)	3 (1–6)	
Unadjusted			1.01 (0.50 to 2.05); *P* = 0.99
Adjusted for baseline^b^			1.01 (0.49 to 2.04); *P* = 0.99
**Duration of any cough (censored at** **56** **days** **)** ^c^	18 (14–31)	13 (6–26)	
Unadjusted			0.68 (0.32 to 1.46); *P* = 0.32
Adjusted for baseline^b^			0.69 (0.32 to 1.48); *P* = 0.35
**Duration of abnormal peak flow** ^c^	24 (4–n/a)^d^	6 (4–n/a)^d^	
Unadjusted			0.45 (0.17 to 1.24); *P* = 0.12
Adjusted for baseline^a^			0.51 (0.18 to 1.42); *P* = 0.21
Adjusted for baseline^e^			0.79 (0.22 to 2.79); *P* = 0.71
	***n* (%)** ****		**OR** **(** **95%** **CI** **);** ***P*** **value**
**Consumption of antibiotics up to** **7** **days**	1 (4.8)	1 (6.3)	
Unadjusted			0.75 (0.04 to 12.99); *P* = 0.84
Adjusted for delayed script			0.74 (0.04 to 13.48); *P* = 0.84
**Consumption of antibiotics up to** **28** **days** ^f^	2 (16.7)	3 (33.3)	
Unadjusted			0.46 (0.07 to 3.12); *P* = 0.42
Adjusted for delayed script			0.38 (0.04 to 3.27); *P* = 0.38
**Participant agrees trial tablets helped them feel better** ^f^	6 (33.3)	6 (40.0)	
Unadjusted			0.75 (0.18 to 3.11); *P*= 0.69
**Participant agrees they would take trial tablets in future** ^f^	9 (50.0)	9 (60.0)	
Unadjusted			0.67 (0.17 to 2.67); *P* = 0.57
**Adverse events** ^g^			
01>1	16 (72.7)6 (27.3)0 (0.0)	14 (77.8)4 (22.2)0 (0.0)	
Unadjusted			1.31 (0.31 to 5.62); *P* = 0.72^g^
Adjusted for baseline^h^			1.17 (0.25 to 5.38); *P* = 0.84^g^

^a^Adjusted for prior duration of cough in days. ^b^Adjusted for presence of symptom at baseline (including previous 24 hours). ^c^Analysis of duration of moderately bad or worse cough (56 days) includes 17 in the prednisolone and 14 in the placebo group (participants without moderately bad or worse cough on day 1 excluded); analysis of duration of any cough (56 days) includes 21 in the prednisolone group and 16 in the placebo group; and analysis of duration of abnormal peak flow includes 11 in the prednisolone group and 13 in the placebo group (participants with normal peak flow on day 1 excluded). ^d^A large proportion of participants still had abnormal peak flow at 28 days – a 75th percentile could therefore not be calculated. ^e^Adjusted for baseline cough duration and baseline abnormal peak flow. ^f^Antibiotic analysis includes 12 in the prednisolone and 9 in the placebo group up to 28 days; patient satisfaction analysis includes 18 in the prednisolone group ad 15 in the placebo group. ^g^Ordinal logistic regression; adverse event data available for 22 in the prednisolone group and 18 in the placebo group. ^h^Adjusted for impression of illness severity. AUC = area under the curve. n/a = not available. SD = standard deviation.

## Discussion

### Summary

This exploratory analysis of the OSAC trial provides evidence that a moderate-dose oral prednisolone does not reduce the duration of moderately bad or worse cough, or symptom severity at days 2–4 in patients presenting to primary care with ALRTI with probable clinically unrecognised asthma.

### Strengths and limitations

The main strength is that, to the authors' knowledge, this is the first time a rigorously conducted, randomised controlled trial has been used to explore the role of corticosteroids for patients with ALRTI and previously unrecognised asthma. A limitation was that the original OSAC study excluded patients who received asthma medication in the last 5 years, meaning it was not possible to include patients with recognised asthma. It is, however, an underdiagnosed condition in adults and older people.^14–18^ Furthermore, the pre-determined definition used to identify unrecognised patients with asthma in this analysis was formulated using strict criteria, using BTS guidelines, and symptom combinations with high sensitivity or specificity for asthma.^10,21^

As the OSAC study questionnaire stated that *‘* *patients must think about symptoms in the* *12* *months* *before their current illness started* *’*,^13^ it is possible that a minority may have misinterpreted this question and included symptoms related to their current ALRTI, resulting in a higher number of patients meeting the criteria for clinically unrecognised asthma. The use of a questionnaire and self-reported outcomes is, in itself, a limitation of the OSAC trial as a whole.

Given that the definition for clinically unrecognised asthma needed to be stringent (while also replicating asthma prevalence in the general population); it was recognised that only a small proportion of the OSAC cohort could be included. Consequently, baseline characteristics were imbalanced; although, additional adjustments were made to account for this. However, despite the relatively low number of patients included in the main analyses, the 95% CIs did not include the pre-specified MCIDs for both primary outcomes.

### Comparison with existing literature

These findings are consistent with the original OSAC trial,^13^ which to the authors’ knowledge is the only study to investigate the benefit of oral corticosteroids in ALRTI. The findings also replicate other studies that have found little benefit from oral or intranasal steroids in acute rhinosinusitis,^26,27^ but contrast with recent meta-analyses demonstrating clinical benefits for sore throat in primary care and community-acquired pneumonia treated in hospital.^11,28^ In the current analysis, the median duration of cough in both placebo and prednisolone groups was 3 days, which was shorter than 5 days observed for both arms in the original OSAC trial. Mean symptom severity scores were also lower in this subset compared with the whole OSAC cohort (1.83 versus 1.99 for prednisolone; 1.95 versus 2.16 for placebo). This was unexpected given that ALRTIs are reported to be more severe and prolonged in people with asthma,^7,8^ and may be owing to chance or patient selection.

### Implications for research and practice

The main reason for conducting this analysis was to see if participants with unrecognised asthma experienced a better response to oral corticosteroids than the remainder of the study sample. This analysis, although underpowered, found no evidence to suggest that they did. If they had, it would have been important for two reasons. First, primary care clinicians might have wished to use the questionnaire (used to identify those with unrecognised asthma) to identify those who would benefit the most, and second, it would have provided an important ‘signal’ that the research community might have wished to confirm in future research.

No evidence was found to support the use of corticosteroids for ALRTI in patients with clinically unrecognised asthma. Clinicians should not use the IPCAG questions to target oral corticosteroid treatment in patients with ALRTI.
